# Professional Development of Teacher Trainers: The Role of Teaching Skills and Knowledge

**DOI:** 10.3389/fpsyg.2022.943851

**Published:** 2022-06-28

**Authors:** Hang Su, Jialin Wang

**Affiliations:** School of Marxism, Dalian Maritime University, Dalian, China

**Keywords:** knowledge, professional development, teacher trainers, teaching skills, teaching knowledge

## Abstract

Since the 1990s, the essential function of teacher trainers in academic courses has gradually attained more attention from scholars. Also, the teacher trainers’ professional development has acquired worldwide attraction following the concept that teacher trainers are deeply liable for educator education quality. The present mini-review of literature indicates that while teacher trainers have several complicated functions, they obtain the least preparation or opportunities for professional development to perform such functions. Consequently, they require getting the related knowledge and skills after accepting the role of teacher trainers. Besides numerous aspects affecting teacher trainers’ professional development, teaching skills, and knowledge have important functions that are at the center of attention in this mini-review of literature. In brief, several implications are presented for the instructional addressees.

## Introduction

The important function of pre-service and in-service educator training platforms in providing educators is a controversial topic in educator training literature (Smith, 2010). Within such programs, educators take the primary measures toward being specialists, achieve higher confidence in their education, and expand the scope of their knowledge reservoir (Akbari and Dadvand, 2011). The teaching job demands ongoing education and growth since it is directly involved with human capital (Harris and Jones, 2010). Educator quality needs educators to have the knowledge and skills in the field they instruct. Educators obtain these skills during their program (Blank and Alas, 2010; Butler, 2015) through Professional Development (PD) which has a vital function in an educator’s future profess and development. Educators require chances to upsurge their knowledge and skills, maintain their incentives, and expand their cooperation with others in their careers (Margolis, 2008). In the history of academia, no attempt at advancement has ever been effective and successful without carefully arranged and well-executed PD actions planned to improve teachers’ knowledge and skills (Guskey, 2009).

The literature on learning has taken part in discussions for years on whether educator standard is the most significant school factor affecting learners’ success and enhancing the standard of the school (Kang et al., 2013; Macia and García, 2016). Similarly, academic leaders, theorists, and scholars have emphasized how to best improve the standard of teaching to enhance learners’ education and success. Each year, nations spend billions of dollars on enhancing the standard of their educators’ skills and eligibilities by building their chances for professional development (PD) (DeMonte, 2013). The standard of teacher education has been known as a central issue influencing the standard of teaching and learners’ success. Therefore, there has been increasing attention to teacher trainers: their individuality, skills, functions, and PD (Loughran, 2014; Lunenberg et al., 2014).

Until recently, teacher trainers were characterized as concealed experts who are not always presented with the help and challenge they require, for instance concerning their learning and PD (Livingston, 2014). Teacher trainers are among those who are engaged in the learning of learners, educators, and ongoing PD of in-service educators (Czerniawski et al., 2017). Nonetheless, in the last 20 years, scholars, and teacher trainers themselves began to growingly notice the particular quality of their jobs, and so, they have begun to emphasize teacher trainers’ PD (Berry, 2016; Lunenberg et al., 2017).

The teacher trainer is considered the most affecting factor when preparing better-organized educators (Snoek et al., 2010), and their function can be explained as a facilitator who links the distance among highpoint level policymakers at the countrywide and/or nearby area. Consequently, they must satisfy the knowledge and function criteria set by employing political organizations and practically display those criteria (Lunenberg et al., 2017). Educator trainers have numerous roles, which need PD and learning (Swennen et al., 2010; Lunenberg et al., 2014) and professional educators should be able to grow knowledge in making well-informed choices regarding activities with approaches that can respond to complicated conditions according to complicated knowledge and reflection (Loughran and Hamilton, 2016).

Classes today have altered with time due to higher levels of diversities and have become more intricate as a result of technology and the generational gap (Gomes et al., 2015; Sonmark et al., 2017). Thus, an educational space is a place for learners to attain novel knowledge and skills and a workplace where educators can study and enhance their careers. And EFL educators necessary need to gain the most recent education knowledge and skills within the milieu of English language education and learning to enhance the learners’ development and growth and perform the international necessities of the universal time (Zhiyong et al., 2020). To comprehend and assist educator trainers’ PD in the best way, it’s vital to understand what skills and knowledge they require and how they efficiently obtain such skills and knowledge during their profession (MacPhail et al., 2019).

Just as the excellence of teachers influences the learning results of students, the eminence of teacher educators impacts the quality of teachers (Darling-Hammond, 2010). The study was done by Buchberger et al. (2000) regarding the growth and the future of teacher education, in which the authors declared that development in the proficiencies of teacher educators may well contribute to significant growth in the quality of teachers. Despite their crucial function in the training and assisting of future educators, research literature and documents on who educator trainers are and how they professionally influence education are not examined until recently (Czerniawski et al., 2017). Overall, presently, there appears to be an agreement that teacher educators are a significant element in deciding the standard of educators, who, in turn, are a significant element in deciding the standard of the education of their learners at different levels of education (Murray and Kosnik, 2011). The professional development literature review indicated that educators’ PD activities mostly reveal their restrained obtaining of knowledge and skills. The issue is caused by the providers’ inability to design PD applications that address the educators’ needs (Darling-Hammond, 2010). Teacher trainers are sometimes held accountable for their vagueness in determining the action purpose and theory in their PD program, however, educators in some other cases are even unwilling to be responsible for their own PD (Daniel and Peercy, 2014). Nonetheless, based on the literature when perspectives from educators merge with that of educator trainers, it can probably ascertain congruence between the contents of educator training and the educators’ needs in addition to higher facilitation of educators’ PD (He et al., 2011). However, based on the researcher’s knowledge, on the one hand, not enough consideration has been paid to teacher educator studies (Lunenberg et al., 2014; Van der Klink et al., 2017) and on the other hand, there is little information regarding educator trainers and their PD: how they are educated and taught and what leads to a proper teacher trainer (Villegas-Reimers, 2003; Lin, 2013).

## Review of the Literature

### Professional Development of Teacher Trainers

Professional development (PD) has been characterized as an inner cycle in which experts are involved in a formal or informal model embedded in the precarious assessment of expert practice (Smith, 2010). Professional development alludes to these types of elevations in knowledge and skills. It is considered the foundation of expert practice in all careers. Villegas-Reimers (2003) argued that the PD of teacher trainers is not famous in comparison with educator PD. Research has suggested that educator PD is one of the impactful elements in learners’ education and success (Villegas-Reimers, 2003; Darling-Hammond, 2010). Professional development is specifically essential for novice educators who must become accustomed to the standards of their careers. Certainly, as stated by Futernick (2007), educators who quit this job regularly state the absence of PD as one of the reasons. PD can also enable educators’ exposure to growing leadership roles. This is particularly crucial to educators in the final level of their profession, whose devotion and inspiration may be falling (Day and Gu, 2007).

Teacher trainers’ PD has been characterized as formal educational and expert progress classes to prepare teachers with pertinent and updated knowledge and capabilities crucial to standard improvement (Sierra-Piedrahita, 2007). In addition, teacher trainers’ PD has been explained as the growth of a question as a position, which alludes to the cycle of ongoing and structured questions wherein they contemplate their own and other’s presumptions and develop local and public knowledge that is suitable for the altering settings in which they work (Loughran, 2014). The aim of teacher educator PD is four-fold: enhancing teacher education, satisfying outer requirements, and inner zeal for studying, enhancing, and fortifying the expert position within higher education (Smith, 2010).

### Knowledge and Skills

Knowledge pertains to the collective term for notions, fundamentals, and activities in a particular area of professional expertise and the overall information, and experience that are critical to efficient functioning in learning and using what is taught (Sierra-Piedrahita, 2007). Pedagogical knowledge is important for educators since it portrays the body of knowledge on educational cycles and settings for learners (O’Riordan, 2018). Alternatively, skills allude to the things “people know how to fulfill” and which are “achieved through practicing” (Sierra-Piedrahita, 2007). Skill or ability refers to the people’s capability to do numerous tasks in a profession and it is also defined by Khorasgani (2019) as something one is familiar with how to perform. Having attempted to designate the knowledge base of education, seven classes of educators’ knowledge are recommended which encompass material knowledge, overall educational knowledge, curriculum knowledge, educational material knowledge, knowledge of learners and their features, knowledge of scholastic settings, knowledge of academic goals, goals, and principles, and their theoretical basis (Ingvarson et al., 2005). Such scope of knowledge is highly difficult for educator trainers and learners similar, because ethnicity, social status, cultural variations, and inequality are sensitive, filled with sense and affection, and links to everybody’s central ideas and values (Goodwin and Kosnik, 2013).

## Conclusion and Implications

Professional development for educators is now deemed as a crucial element of guidelines to improve the standard of teaching and education in colleges. Therefore, there is prominent attention to studies that determine attributes of successful professional education (Ingvarson et al., 2005). The educational intention of the PD for educators was to attain the skills required to enable learners’ education through explorations that aimed at scientific inquiry skills covered in their teaching process. However, teacher trainers are being considered professionals and their PD is inevitably on the rise. Teacher educators’ PD is an inevitable cycle and a crucial component of enhancing learning overall; therefore, teacher trainers should be dynamic mediators in their growth by keeping themselves up to date with novel information developing and improving knowledge on education and teacher instruction to enhance and boost their own teaching.

In addition, educator trainers need to teach educators with enough knowledge of learners’ learning patterns and tactics. Educators require learning regarding various methods of learning as employed by different individuals, such that they can efficiently goal education in the direction of learners’ learning requirements. Education knowledge and skills are anticipated to be designed by educator trainers during their educator training classes as they are typically liable to make them explicit and reachable to learner educators. Teacher trainers are predicted to build novel knowledge, including knowledge in practice in the framework of recent curricula and learning programs for educator trainers and schools besides knowledge in theory produced from studies.

Based on the literature, an effective teacher educator needs to have enough knowledge of particular and efficient approaches to expose scholar-teachers to numerous diverse techniques of teaching and they are also capable of assisting them to collect a remarkable style of their own teaching. Thus, educator trainers must get acquainted with the knowledge of research and skills, and with the skills to monitor learner educators in doing studies. Professional development practices were made to make them ready for it. Teacher educators feel better equipped for the new tasks if they are provided with the prospects to be present at PD tasks with the accurate situations. There is a need to hold seminars and conferences as they are the main paths to PD for educator trainers and they are sometimes employed for bringing in new knowledge and activity. More studies specifically empirical should be conducted that employ interview as through implementing the interviews, more in-depth understanding can be achieved.

## Author Contributions

Both authors listed have made a substantial, direct, and intellectual contribution to the work, and approved it for publication.

## Conflict of Interest

The authors declare that the research was conducted in the absence of any commercial or financial relationships that could be construed as a potential conflict of interest.

## Publisher’s Note

All claims expressed in this article are solely those of the authors and do not necessarily represent those of their affiliated organizations, or those of the publisher, the editors and the reviewers. Any product that may be evaluated in this article, or claim that may be made by its manufacturer, is not guaranteed or endorsed by the publisher.
